# Outbreak of central nervous system infections among children in Thai Binh, Viet Nam

**DOI:** 10.1080/22221751.2022.2088405

**Published:** 2022-06-20

**Authors:** Duc Long Phi, Xuan Duong Tran, Minh Manh To, Hai Yen Dang, Thi Dung Pham, Thi Thu Trang Vu, Trong Kiem Tran, Manh Dung Do, Thi Thuy Vu, Stéphane Ranque, Laetitia Ninove, Sylvie Pillet, Philippe Colson, Bernard La Scola, Van Thuan Hoang, Philippe Gautret

**Affiliations:** a Thai Binh University of Medicine and Pharmacy, Thai Binh, Viet Nam; b IHU-Méditerranée Infection, Marseille, France; c Aix Marseille Univ, IRD, AP-HM, SSA, VITROME, Marseille, France; d Thai Binh Pediatric Hospital, Thai Binh, Viet Nam; e Unité des Virus Émergents (UVE: Aix-Marseille Univ – IRD 190 – Inserm 1207 – IHU Méditerranée Infection), Marseille, France; f Laboratoire des agents infectieux et d'hygiène, CHU de Saint-Étienne, France; g CIRI- International Center of Research in Infectiology, Centre International de Recherche en Infectiologie, GIMAP Team University of Lyon, University of St-Etienne, INSERM U1111, CNRS UMR5308, ENS Lyon, Claude Bernard Lyon 1 University, Lyon, France; h Aix Marseille Univ, IRD, AP-HM, MEPHI, Marseille, France

**Keywords:** Central nervous system, meningitis, children, enterovirus, Thai Binh

## Abstract

From July to October 2020, 99 cases of central nervous system (CNS) infections were identified in Thai Binh Pediatric Hospital, Viet Nam, representing a five-fold increase compared to the baseline incidence during the previous five years. Clinical data were retrospectively collected. Cerebrospinal fluid specimens (CSF) were secondarily tested for pathogens using viral culture and PCR assays. Patient median age was 5 years (0–12 years); 58.6% were male. Of these children, 83.8% had CSF white blood culture (WBC) counts of ≥ 10 cells/µL, including 58 of 99 (58.6%) with a WBC count ≥ 100 cells/µL. Overall, 72 (72.7%) patients had confirmed infections with a pathogen identified in the CSF, the majority of which (66) were enterovirus. Sequencing results suggested that the rise of incidence observed in 2020 was due to Echovirus 4 (*n* =  45), Echovirus 30 (*n* =  8), and Echovirus 6 (*n *=  1) circulation. A confirmed CNS infection was significantly associated with older age (≥5 years, OR = 3.64, *p* = 0.03) and with an increased WBC count in the CSF (OR = 6.38, *p*-value = 0.01 for WBCs from 10 to <100 and OR = 7.90, *p*-value = 0.002 for WBCs ≥100). Ninety-seven (97) of 99 (98.0%) children received empiric antimicrobial treatment, and 35 (35.3%) were treated with multiple antibiotics. Eighty-four (84) patients (84.9%) were discharged home, and 11 (11.1%) were transferred to the National Hospital because their condition had worsened. No deaths were recorded. Point-of-care tests, including real-time PCR assays to identify common pathogens, should be implemented for more accurate diagnosis and more appropriate antibiotic use.

## Introduction

Central nervous system (CNS) infections pose a major problem for public health. These infections, including meningitis, encephalitis, and brain abscesses, are potentially life-threatening conditions, particularly among young children [1]. In addition to high mortality rates, these diseases also cause serious irreversible complications. CNS infections rank 6th (2.2%) of all causes of death in children under 5 years old and 8th (3.4%) in children aged 5–14 years [1]. The incidence and mortality rate of CNS infections are considerably higher in low- and middle-income countries as compared to high-income countries [https://vizhub.healthdata.org/gbd-compare/].

Rapid and accurate identification of microbial pathogens is essential in the management of CNS infections. Common methods used in microbiology laboratories are antigen or antibody detection, direct microscopic examination, and culture techniques. But these methods are limited by low sensitivity and/or specificity. Currently, with high accuracy and short execution time, molecular methods performed on cerebrospinal fluid (CSF) samples are considered the “platinum” standard, in contrast to the culture “gold” standard in the etiological diagnosis of CNS infections [2]. However, the molecular test is not always available in developing countries.

Several infectious pathogens are known to cause CNS infection, including the broad categories of viruses, bacteria, mycobacteria, fungi, and parasites. In children, viruses are the more common pathogens responsible for CNS infections, which are mostly mild and self-limited [2]. Bacterial CNS infections are often severe and continue to be a challenging disease that needs to be recognized and treated quickly to prevent life-threatening complications [3]. The etiology of CNS infections can vary by geographic region and season [2]. Therefore, epidemiological research on the causes of CNS infections plays an important role in determining the etiology of the disease in order to provide quick diagnostic measures, patient treatment, and reduced testing cost.

**Figure 1.**
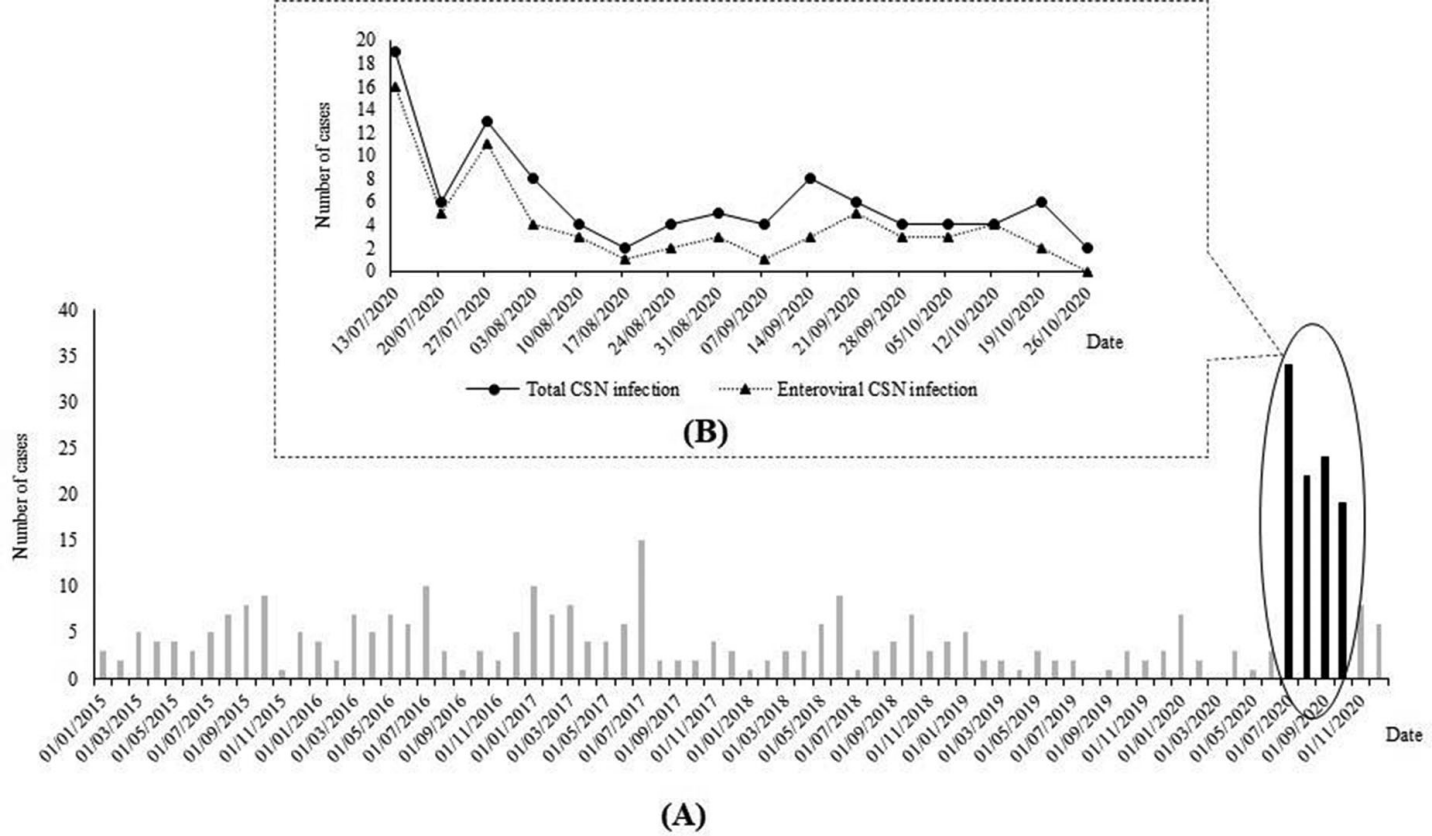
Monthly distribution of CNS infections among children in Thai Binh Pediatric Hospital, Viet Nam from 01/01/2015 to 31/12/2020 (black bars: period of current study) (A) and daily distribution of CNS infections among children in Thai Binh Pediatric Hospital, Viet Nam during the study period (B)

In Thai Binh, Viet Nam, CNS infections, including meningitis and encephalitis, are frequent infectious diseases in children [4]. In our preliminary report, 250 patients with CNS infections were diagnosed over the course of 5 years (2015–2019) in the Thai Binh Pediatric Hospital (TBPH), corresponding to approximately 4 cases per month. Surprisingly, from July to October 2020, about 100 cases were identified, representing a six-fold increase compared to the baseline incidence (Figure 1). This suggested the occurrence of a meningo-encephalitis outbreak. Here we retrospectively studied the clinical and epidemiological data and searched for the pathogens responsible for this outbreak of CNS infections among children in Thai Binh.

## Materials and methods

### Study setting

This study was carried out in TBPH, a large hospital in the central province of Thai Binh. In 2019, this hospital had 565 beds distributed across 15 different medical departments. Ill children under 16 years are directly admitted from home (through a private clinic, general outpatient, or emergency departments) or referred from district hospitals in the province. Children with severe disease who do not improve are transferred to the Vietnam National Children’s Hospital in Hanoi (VNCH). The identification of microorganisms in the laboratory is very limited, relying mainly on common bacterial culture and serological tests [4].

### Study design and case definition

Between July and October 2020, all ill children admitted to the TBPH meeting the clinical case definition for CNS infections (Table 1) [5] were included in this study. Briefly, for study enrolment, four criteria had to be met: age under 16 years, objective fever within 24 h of hospitalization, clinical features with at least one with suggestive signs of CNS infection, and availability of cerebrospinal fluid (CSF) obtained by lumbar puncture (Table 1).

**Table 1.** Clinical definition and classification of CNS infection based on laboratory features of CNS infections among children.

Clinical definition (Included criteria)*	Features
Age	0–16 years
Fever	≥38°C
Clinical features	At least one:
	Stiff neck Altered/reduced consciousness Focal neurological signs Convulsion Bulging fontanelle if <12 months of age Irritability if <5 years of age Headache if <5 years of age Prostration • Petechial or purpuric rash
Laboratory investigation	Lumbar puncture performed, or planned at the time of assessment, by clinical team
CNS infection classification based on laboratory feature	Features (in addition to meeting clinical case definition)
Confirmed	Pathogen detected in CSF by culture and/or PCR OR Positive serology in CSF OR Positive blood culture AND purulent CSF (WBC ≥100 cells/μL OR [WBC 10–99 cells/μL AND glucose <2.2 mmol/L or protein >1.0 g/L]) OR Purulent CSF AND positive CSF Gram stain
Probable	Purulent CSF AND absence of identifiable pathogens by culture, Gram stain, PCR, or serology** OR Abnormal CSF (WBC 10–99 cells/μL AND normal protein/glucose) AND positive blood culture and/or positive CSF Gram stain
Suspected	Non-purulent CSF AND absence of identifiable pathogens by culture, PCR, or serology

Note: *For study enrolment, all four criteria had to be met (age, fever, clinical features, and laboratory).

**Pathogen-negative probable cases were re-categorized as suspected cases if laboratory testing was incomplete.

CNS: central nervous system, CSF: cerebrospinal fluid, WBC: white blood cell, PCR: Polymerase Chain Reaction.

### Clinical data and specimen collection

Clinical data, including sociodemographic characteristics, clinical symptoms, basic laboratory investigation, treatment and outcome were retrospectively obtained from medical electronic records.

Routine laboratory investigations were performed at the discretion of the receiving clinician and based on hospital guidelines. Blood specimens were collected from all patients for white blood cell (WBC) counts and C-reactive protein (CRP) detection. Blood cultures were performed on a small number of patients (*n* = 24). CSF was collected from all patients for standard laboratory testing (cell counts and biochemical markers), standard bacterial culture and anti-JEV (Japanese encephalitis virus) IgM detection in the TBPH hospital laboratory.

In addition, aliquots of CSF were stored at −80°C in a freezer within 1 h of collection and transferred to the Marseille IHU Méditerranée Infection laboratory, France, on dry ice to perform further diagnostic investigations.

### Laboratory assays

#### Routine laboratory investigations in Thai Binh Pediatric Hospital

Due to the lack of microbiological testing facilities, blood culture was performed according to the clinicians’ expertise; usually it is indicated in patients with suspected sepsis. Blood cultures were performed on 2–5 mL venous blood, inoculated into 20 mL culture medium (brain heart infusion broth (Oxoid, Basingstoke, UK)) plus 0.05% sodium polyanethol sulfonate (Sigma-Aldrich, St. Louis, MO, USA). The vented bottles were incubated at 37°C for up to seven days. Bottles were routinely sub-cultured onto solid media at 24 h and seven days, with additional subculture if turbidity was noted on daily inspection.

CSF specimens (2–3 mL) were routinely submitted for red and white blood cell counts (using a Fuchs-Rosenthal counting chamber with a Giemsa-stained slide for differential white blood cell count), glucose and protein estimation (HumaStar 200 analyzer; Human, Wiesbaden, Germany). IgM against Japanese encephalitis virus (JEV) were detected using the commercial kit JE MAC-ELISA (National Institute of Hygiene and Epidemiology, Hanoi, Vietnam) according to the manufacturers’ recommendations.

In addition, CSF was submitted for Gram stain, then cultured onto 5% sheep blood agar, chocolate agar, MacConkey and Sabouraud agar plates (Oxoid, prepared in-house) for up to 48 h. Bacterial isolates from blood and CSF cultures were identified using the VITEK Mass Spectrometry System (Vitek MS; bioMérieux, Durham, NC, USA).

#### Diagnostic techniques performed at IHU Méditerranée Infection, Marseille, France

### Viral culture and identification

For each CSF specimen, 100 µL was passed through a 0.22-μm pore size centrifugal filter (Merck Millipore, Darmstadt, Germany) and then were inoculated into four wells of 96-well culture microplates containing MDCK cells (ATCC CCL-34) in MEM with 10% fetal calf serum and 1% L-glutamine. After centrifugation at 4000×g, microplates were incubated at 37°C. Each sample was cultured until a cytopathic effect (CPE) was observed, usually requiring one to four passages (seven days per passage). Viral growth was detected by searching for CPE daily, using an inverted microscope, then confirmed using a multiplex PCR assay (Fast-Track Diagnosis (FTD, Sliema, Malta)).

### Molecular identification

Molecular identification was first carried out using the BioFire FilmArray Meningitis/Encephalitis Panel (ME). Approximately 200 μL of CSF was subject to FilmArray® ME Panel testing according to the manufacturer’s instructions. The panel identifies 14 common agents of community-acquired meningoencephalitis: *Haemophilus influenzae, Streptococcus pneumoniae, Neisseria meningitidis, Escherichia coli K1, Streptococcus agalactiae, Listeria monocytogenes*, enterovirus (EV), herpes simplex virus type 1 (HSV-1), herpes simplex virus type 2 (HSV-2), human herpesvirus type 6 (HHV-6), cytomegalovirus (CMV), varicella zoster virus (VZV), human parechovirus (HPeV), and *Cryptococcus neoformans/gattii*. This test includes automated sample homogenization, nucleic acid extraction, reverse transcription, nucleic acid amplification, and results in analysis in approximately one hour per run per specimen.

Additional real-time PCR tests were performed to identify JEV, dengue virus, two bacteria (*Klebsiella pneumoniae* (*phoE* gene) and *Staphylococcus aureus* (*nucA* gene)), and five main Candida species (*C. parapsilosis, C. tropicalis, C. albicans, C. glabrata*, and *C. rugosa*) on supernatant after viral culture. These five *Candida* spp. were selected because unlike *Cryptococcus* spp. infections that usually occur in adults and exceptionally in children, *Candida* spp. are agents of neonatal infections (including meningoencephalitis). Their prevalence is probably low but possibly overlooked because they are not routinely diagnosed in Vietnam. The laboratory manipulation procedure and protocol have been described elsewhere [6,7].

Enterovirus genotyping was performed from enterovirus-positive RNA extracts based on partial genome regions of the VP1 capsid protein gene by Sanger population direct sequencing on an ABI3500 instrument (Applied Biosystems, Foster City, USA), according to a previously described procedure [8]. Genotype was determined based on BLAST searches [9] against the NCBI GenBank nucleotide sequence database (https://www.ncbi.nlm.nih.gov/genbank/) [10] or phylogenetic analyses. For phylogeny reconstruction, nucleotide alignments were performed using the ClustalW program with the BioEdit software v7.0.9.0 [11]. The evolutionary history was inferred in the MEGAX v10.2.6 software (http://www.megasoftware.net/) [12] using the Neighbour-Joining method and the Kimura 2-parameter method; this incorporated de-duplicated sequence sets built with the 5 best hits retrieved by BLAST searches [9] from GenBank using each sequence obtained here as queries.

With the aim to detect viruses that were not identified by PCR, to obtain viral genetic material for facilitating sequencing, and to store the viral isolates for any further analyses, CSF samples were inoculated on Vero E6 cells (ATCC CRL-1586) as previously described [13,14]. Next-generation virus genome sequencing was performed from the culture supernatant by Illumina technology on a MiSeq instrument using a procedure previously applied to other viruses [14]. The viral genome was obtained by next-generation sequencing with Illumina technology using the Nextera XT paired end strategy on a MiSeq instrument (Illumina Inc., San Diego, CA, USA). Consensus genomes were assembled by mapping on reference genomes, GenBank accession No. MK815088.1 (for enterovirus genomes), KF268199.1 (for the adenovirus genome), and OK440541.1 (for the mumps virus genome) using the CLC Genomics workbench v.7 (https://digitalinsights.qiagen.com/) as previously described [8,15]. For EV genome analysis, phylogeny reconstruction was performed as sequences obtained by Sanger sequencing except nucleotide alignments were performed using the Mafft online alignment tool (for sequences obtained by next-generation sequencing; https://mafft.cbrc.jp/alignment/server/) [16].

### Data analysis

Data were double-entered using Microsoft Access, cleaned, and exported to STATA software version 14.2 (Copyright 1985–2015 StataCorp LLC, http://www.stata.com) for analysis. Continuous variables were analyzed and expressed as median and ranking. Categorical variables were presented as numbers and proportions. Chi2 or Fisher exact test was used to compare the difference in proportion, when appropriate.

Based on the results of microbiology tests, diagnoses at discharge were classified as confirmed, probable, or suspected CNS infection (Table 1) [5].

Our main predictive factors associated with the detection of a pathogen in CSF samples were categorized as follows: Age (<12 months, 1–4 years, ≥5 years), sex (male, female), clinical presentation, blood test results (WBC count and CRP) and laboratory CSF examination (WBC count, proteinorachia, glycorrhachia).

To investigate the predictive factors, only variables with a prevalence ≥5.0% were introduced into the statistical analysis. Bivariate logistic regression analysis was used to calculate the odd ratios (OR) for the association between the detection of a pathogen in CSF samples and independent variables. Next, multivariate analysis was carried out, adjusted for all variables with *p* values <0.2 in the univariate analysis. Multivariate analysis was performed using exact logistic regression. Adjusted odds ratios (aOR) with a 95% confidence interval (95% CI) and *p*-values <0.05 were used to determine if a predictive factor was statistically significantly associated with the detection of a pathogen in CSF samples.

### Ethics

This was a non-interventional study, requiring no additional procedures or tests for the patient. The study was based on the patient's health data available in their medical records. Therefore, there was no direct benefit to the patient. The protocol was approved by the Ethics Committee of Thai Binh Pediatric Hospital (January 18, 2021; reference No. 2021.046).

## Results

### Sociodemographic characteristics and clinical features of the studied population

In total, 99 patients were eligible for enrolment in our study according to the clinical case definition for CNS infections (Table 2). The median age at admission was 5 years (interquartile [0–8], range = 0–12 years). Half (50 of 99, 50.5%) were children aged from 5 to 16 years, followed by those under 12 months (26 of 99, 26.3%) and those aged 1–4 years (23 of 99, 23.2%). Fifty-eight (58.6%) were male and 41 (41.4%) were female; hence, the sex ratio (M/F) was 1.4. Most children were admitted to TBPH directly from home (75 of 99, 75.8%) and the remaining were transferred from district hospitals (24 of 99, 24.2%). Twelve children (12.1%) received antibiotics before admission.

**Table 2.** Characteristics of included patients.

Characteristics	*N* = 99 *n* (%)
Age	
Median [range]	5 (0–12)
<12 months	26 (26.3)
1–4 years	23 (23.2)
≥ 5 years	50 (50.5)
Gender	
Male	58 (58.6)
Female	41 (41.4)
History	
Fever	99 (100)
Vomiting	52 (52.5)
Diarrhea	2 (2.0)
Reduced feeding/eating & drinking	3 (3.0)
Lethargic	18 (18.2)
Headache (*N *= 50)	46 (92.0)
Seizure	2 (2.0)
Antimicrobial auto-medication prior to admission	12 (12.1)
Symptoms duration before admission (days), median (range)	2 (1–7)
Transfer from district hospitals	24 (24.2)
Physical findings	
Stiff neck	45 (45.4)
Altered/reduced consciousness	3 (3.0)
Focal neurological symptoms	2 (2.0)
Irritability (*N* = 50)	6 (12.0)
Bulging fontanelle (*N* = 26)	23 (88.5)
Purpuric rash	1 (1.0)
Laboratory findings	
Blood examination	
White blood cells	
Normal	5 (5.1)
Elevated CRP	94 (94.9)
Normal	30 (30.3)
Elevated	69 (69.7)
CSF laboratory examination	
WBC	
<10	16 (16.2)
10–<100	25 (25.2)
≥ 100	58 (58.6)
Protein	
≤ 1.0 g/L	87 (87.9)
>1.0 g/L	12 (12.1)
Glucose	
≥ 2.2 mmol/L	94 (95.0)
< 2.2 mmol/L	5 (5.0)

CRP: C-reactive protein; CSF: cerebrospinal fluid; WBC: white blood cell.

Regarding clinical findings, 45 (45.4%) children had stiff neck, 23 of 26 (88.5%) patients aged under 12 months had a bulging fontanelle. Only one patient (1.0%) presented with a purpuric rash (Table 2).

Blood examination showed that 94 (94.9%) and 69 (69.7%) children had elevated WBC counts and elevated CRP levels, respectively. CSF laboratory examination showed that 83 of 99 (83.8%) children had WBC count of ≥ 10 cells/µL, including 58 of 99 (58.6%) with WBC count ≥ 100 cells/µL. A total of 12 (12.1%) and 5 (5.0%) children had hyper-proteinorachia (>1.0 g/L) and hypo-glycorrhachia (<2.2 mmol/L) (Table 2).

### Pathogen detection (Table 3)

In TBPH, all patients were tested for JEV IgM and underwent CSF culture, but no case was positive. In addition, 24 children underwent blood culture. Of those, five were positive for *Staphylococcus aureus* (2), *Streptococcus agalactiae* (1), *Burkholderia cepacia* (1) and *C. parapsilosis* (1).

**Table 3.** Pathogens identified in 99 included children.^1^

Pathogens	Viral culture *n* (%)	BioFire multiplex PCR* n (%)	Overall *n* (%)
CSF			
Enterovirus	11 (11.1)	62 (62.6)	66 (66.7)
Adenovirus	1 (1.0)		1 (1.0)
Enterovirus and mumps virus coinfection	1 (1.0)	–	1 (1.0)
Herpes simplex virus 1	0 (0)	1 (1.0)	1 (1.0)
Herpes simplex virus 6	0 (0)	1 (1.0)	1 (1.0)
*Haemophilus influenzae*	–	1 (1.0)	1 (1.0)
*H. influenzae* and *Streptococcus pneumoniae* coinfection	–	1 (1.0)	1 (1.0)
At least one pathogen	13 (13.1)	66 (66.7)	72 (72.7)
Blood culture			
*Burkholderia cepacia*	–	–	1 (1.0)
*Candida parapsilosis*	–	–	1 (1.0)
*Staphylococcus aureus*	–	–	2 (2.0)
*Streptococcus agalactiae*	–	–	1 (1.0)
At least one pathogen	–	–	5 (5.1)

Notes: *No sample was positive for *Neisseria meningitidis, Escherichia coli K1, S. agalactiae, Listeria monocytogenes*, herpes simplex virus type 2, cytomegalovirus, varicella zoster virus, human parechovirus, and *Cryptococcus neoformans/gattii*.

^1^ Additional real-time PCR tests were performed to identify Dengue virus, two bacteria: (*Klebsiella pneumoniae* and *Staphylococcus aureus*), and five main Candida species (*C. parapsilosis, C. tropicalis, C. albicans, C. glabrata*, and *C. rugosa*) on supernatant after viral culture. But no sample was positive for these pathogens.

CSF: cerebrospinal fluid; PCR: Polymerase Chain Reaction.

Thirteen patients (13.1%) had a positive viral culture for EV (11, 11.1%), adenovirus (1, 1.0%) and both EV and mumps virus (1, 1.0%).

The BioFire multiplex PCR test was positive for at least one pathogen in 66 (66.7%) patients. Most patients (62 of 99, 66.7%) were positive for enterovirus. Only four (4.0%) patients were positive for four other pathogens (Herpes simplex virus 1 (1), Herpes simplex 6 (1), *H. influenzae* (1) and *H. influenzae-S. pneumoniae* co-infection (1)).

Overall, 72 (72.7%) patients were positive for at least one pathogen in the CSF. Of those, 66 (66.7%) were EV-associated CNS infections, and these cases were distributed throughout the study period, in parallel with the total number of cases (Figure 1).

Regarding the five patients with a positive blood culture, only one (with *S. agalactiae* positive blood culture) was also positive for pathogen detection in the CSF (Herpes simplex virus 1). No pathogen was identified in the CSF of the four remaining patients.

### Viral sequencing and culture from positive CSF

**Figure 2.**
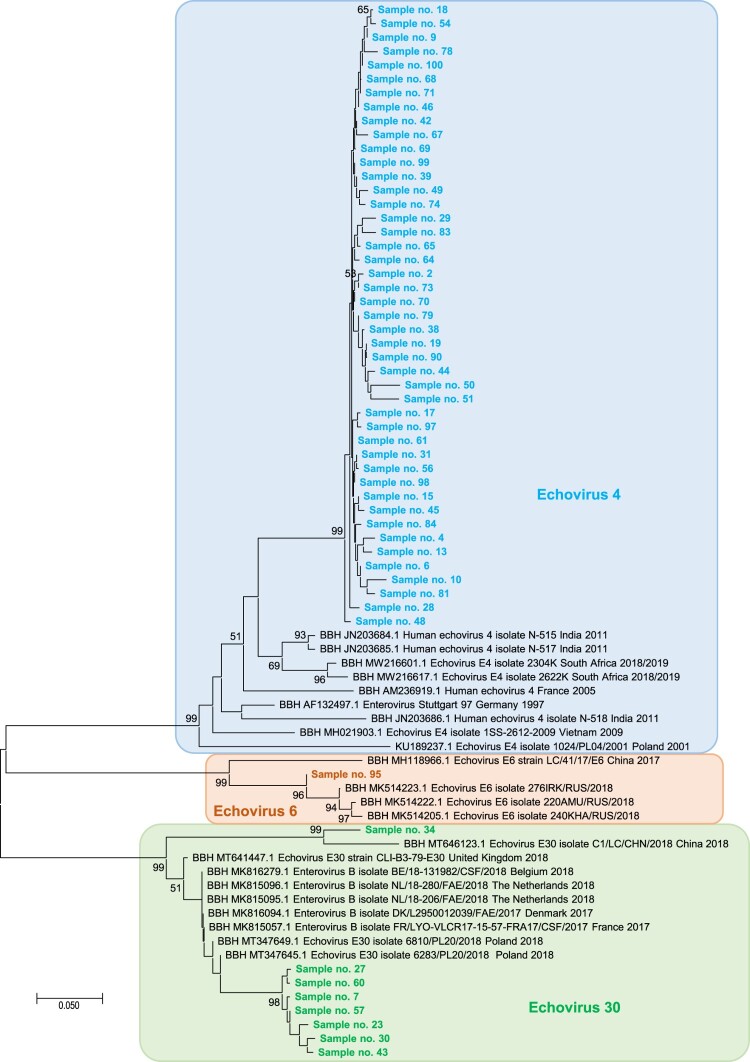
Phylogenetic tree based on partial VP1 gene sequences obtained in the present study. The sequences with the highest BLAST scores recovered from the NCBI GenBank nucleotide sequence database (http://www.ncbi.nlm.nih.gov/nucleotide/), indicated by BBH for best BLAST hit, for each sequence obtained here (indicated by a label starting by “Sample no.”) were incorporated in the phylogeny reconstruction. Alignment positions corresponded to coordinates 2462–2636 of sequence GenBank accession no. AF132497.1. Nucleotide alignments were performed using the ClustalW program with the BioEdit software v7.0.9.0 [11], and the evolutionary history was inferred in the MEGAX v10.2.6 software (http://www.megasoftware.net/) using the Neighbour-Joining method and the Kimura 2-parameter method. The percentage of replicate trees in which the associated taxa clustered together in the bootstrap test (1000 replicates) is shown next to the branches. The tree is drawn to scale, with branch lengths in the same units as those of the evolutionary distances used to infer the phylogenetic tree. The scale bars indicate the number of nucleotide substitutions per site. Bootstra*p* values >50% are labelled on the tree.

Among the 66 samples positives for an EV, we identified three different Echovirus (E) types, including E-4 (in 45 cases), E-30 (in 8 cases), and E-6 (in 1 case), based on BLAST searches [9] against the NCBI GenBank nucleotide sequence database (nt) or phylogenetic analyses using four different partial VP1 gene sequences (Figure 2). This indicated that the rise of CNS infections in Thai Binh in 2020 were due to different epidemics. For E-4 sequences obtained here, mean (± standard deviation) nucleotide similarity was 94.8 ± 2.2% between each other and all sequences were clustered together, apart from their best hits, with which they showed a lower mean of maximum similarities (89.0 ± 1.5%). Best hits had been obtained from samples collected in India, South Africa, France, Germany, Vietnam, and Poland between 1997 and 2018–2019 (Figure 2). For Echovirus 30, mean similarity was 92.5 ± 8.0%. These sequences were clustered (mean of maximum similarities, 95.3 ± 1.5%) with sequences originating from samples collected in China, United Kingdom, Belgium, the Netherlands, Denmark, France, and Poland between 2017 and 2018. Finally, the E-6 sequence showed a maximum similarity of 95.4% with sequences originating from samples collected in China and Russia in 2017–2018.

A cytopathic effect was observed for seven samples positive by qPCR for EV, adenovirus, and mumps virus (in 6, 2 and 1 cases, respectively; one sample was positive for an EV and an adenovirus). Six echovirus genomes, an Adenovirus C genome and a mumps virus genome were obtained from 6, 1 and 1 CSF, respectively. They were compared to their 20 best BLAST hits collected from GenBank (https://www.ncbi.nlm.nih.gov/genbank/). The echovirus genomes were clustered in the phylogenetic tree with E-30 genomes (Supplementary Figure S1), but the mosaicism of the EV genomes makes tricky their classification as the result can differ according to the genomic region considered. The 6 genomes were clustered together apart from their best BLAST hits from GenBank that originated from Austria, Ireland, and the Netherlands. Five of the six genomes shared a mean nucleotide identity of 94.9 ± 5.9%, whereas the sixth genome was more distantly related, showing a mean identity of 83.4 ± 1.1%. Regarding the adenovirus C genome, the most similar genomes have been obtained in China, Switzerland, and the USA (data not shown). Regarding the mumps virus genome, the most similar genome was that of the L-Zagreb vaccine strain (AY685920.1) (data not shown).

### Clinical presentation among patients infected with different pathogens

We compared the clinical presentation of patients infected with Echovirus 4 versus the remaining patients. The results are showed in supplementary table 1. Patients infected with Echovirus 4 were more likely to be aged ≥5 years and reporting vomiting. They were also more likely to present with high numbers of white blood cells in the CSF.

### Diagnosis at discharge of included patients

Based on laboratory criteria, 72 (72.7%) children were classified as confirmed CNS infection, 14 (14.2%) as probable infection and the remaining 13 (13.1%) patients were diagnosed as suspected CNS infection.

### Factors associated with the detection of a pathogen in CSF samples (Table 4)

Univariate analysis showed that patients aged ≥5 years were significantly more likely to have a pathogen identified in CSF compared with those under 12 months (OR = 5.25, *p*-value = 0.003). Detection of a pathogen in CSF samples tends to occur less frequently in boys than in girls (OR = 0.39, *p*-value = 0.06), and to be associated with vomiting (OR = 2.38, *p*-value = 0.06). WBC counts in CSF from 10 to <100 and ≥ 100 cells were significantly associated with confirmed CNS infection (OR = 6.97, *p*-value = 0.007 and OR = 10.56, *p*-value <0.001, respectively).

**Table 4.** Univariate and multivariate analysis of potential factors associated with the detection of a pathogen in CSF samples.

Variable	Confirmed CNS infection (*N* = 99) *n* (%)	Univariate analysis		Multivariate analysis	
		OR [95%CI]	*p*-value	OR [95%CI]	*p*-value
Age					
<12 months	13 (50.0)	ref	ref	ref	ref
1 - 4 years	17 (73.9)	2.83 [0.85–9.47]	0.09	1.74 [0.45–6.74]	0.42
≥ 5 years	42 (84.0)	5.25 [1.79–15.43]	0.003	3.64 [1.11–11.95]	0.03
Gender					
Female	34 (82.9)	ref	ref	ref	ref
Male	38 (65.5)	0.39 [0.12–1.12]	0.06	–	–
Vomiting					
No	30 (63.8)	2.38 [0.96–5.92]	0.06	–	–
Yes	42 (80.8)				
Lethargic					
No	60 (74.1)	0.70 [0.23–2.10]	0.53	–	–
Yes	12 (66.7)				
Stiff neck					
No	37 (68.5)	1.61 [0.65–3.99]	0.31	–	–
Yes	35 (77.8)				
Increase in WBC					
No	28 (80.0)	0.55 [0.21–1.47]	0.23	–	–
Yes	44 (68.8)				
Increase in CRP					
No	22 (73.3)	0.96 [0.36–2.52]	0.93	–	–
Yes	50 (72.5)				
WBC in CSF					
< 10	5 (31.3)	ref	ref	ref	ref
10 - <100	19 (76.0)	6.97 [1.72–28.25]	0.007	6.38 [1.49–27.24]	0.01
≥ 100	48 (82.8)	10.56 [3.00–37.14]	<0.001	7.90 [2.07–30.13]	0.002
Increase of proteinorachia					
No	65 (74.7)	0.47 [0.14–1.65]	0.24	–	–
Yes	7 (58.3)				
Decrease of glycorrhachia					
No	70 (74.5)	0.23 [0.04–1.45]	0.12	–	–
Yes	2 (40.0)				

CRP: C-reactive protein; CSF: cerebrospinal fluid; WBC: white blood cell.

In multivariate analysis, confirmed CNS infection remained significantly associated with older age (≥5 years, adjusted OR = 3.64, *p*-value = 0.03) and with increased WBC count in CSF (adjusted OR = 6.38, *p*-value = 0.01 for WBC from 10 to <100 and adjusted OR = 7.90, *p*-value = 0.002 for WBC ≥100).

### Patient treatment and outcomes (Table 5)

Ninety-seven of 99 (98.0%) children received empiric antimicrobial treatment. Of those, 35 (35.3%) were treated with multiple antibiotics. Cephalosporins and aminoglycosides were most frequently used in 88 of 99 (88.9%) and 21 of 99 (21.2%) cases, respectively. One patient with blood culture positive for *C. parapsilosis* received amphotericin B. The duration of antimicrobial treatment varied from 1 to 39 days. It should be noted that two patients were not treated with antibiotics because they were discharged against medical advice. In addition, 60 (60.6%) children received corticosteroids.

**Table 5.** Treatment and outcomes of children admitted for central nervous system infection.

Characteristics	*N* = 99 *n* (%)
Empiric antimicrobial treatment	
Yes	97 (98.0)
No	2 (2.0)
Multiple empiric antibiotics	35 (35.3)
Beta-lactam	17 (17.2)
Cephalosporin	88 (88.9)
Carbapenem	6 (6.1)
Aminoglycosides	21 (21.2)
Glycopeptide	1 (1.0)
Antifungal	1 (1.0)
Duration (days), median (range)	9 (1–39)
Corticosteroids	
Yes	60 (60.6)
No	39 (39.4)
Hospitalization duration (days), median (range)	10 (1–40)
Outcomes	
Discharge	84 (84.9)
Transfer to national hospital	11 (11.1)
Discharge against medical advice	4 (4.0)
Death	0 (0)

A total of 84 patients (84.9%) were discharged home, and 11 (11.1%) were transferred to the Viet Nam National Children Hospital because their condition had worsened. Four patients were discharged against medical advice. No deaths were recorded.

## Discussion

We describe the epidemiology and etiology of an outbreak of CNS infections among children in Thai Binh. We isolated a pathogen in 72.7% of cases, which is higher than in previous studies in Vietnam and neighbouring countries [3,17,18]. In a prospective provincial hospital-based descriptive surveillance study conducted in 1241 Vietnamese patients, etiologies were diagnosed in 52% of cases [3]. Infectious pathogens were identified in 98 of 149 (65.8%) patients with meningoencephalitis in Thailand from 2003 to 2005 [17]. Among children, Horwood et al. conducted a study in Cambodia, and microbiological etiologies were found in 44.2% of patients with meningoencephalitis [18]. A Chinese multicenter prospective study conducted in paediatric patients with encephalitis (261) and meningitis (285) showed that the etiology was detected in 52.5% and 42.8% of patients, respectively [19]. The high percentage of confirmed CNS infection that we found in this study could be explained by the occurrence of an outbreak of EV of different genotypes. These viruses can be easily detected using the BioFire assay, and outbreaks of easily detectable pathogens would always lead to a higher percentage of detected disease.

JEV is one of the most frequent pathogens responsible for CNS infection in Asian tropical countries, including Vietnam [5,20,21], but no case was positive for JEV in our study. This can be explained because the vaccine against JEV has been introduced into the immunization programme in Vietnam since 1997. Unfortunately, in this study, we did not have data on the Japanese encephalitis vaccination status among included patients, but in Vietnam overall, more than 90% of children between one to five years old at high risk of Japanese encephalitis received three doses of JEV vaccine [https://www.ngocentre.org.vn/news/vietnam-launch-nationwide-japan-encephalitis-vaccination-amid-high-risks]. In a national acute encephalitis syndrome surveillance study conducted from 1998 to 2007, the annual incidence of encephalitis in Vietnam ranged from 3.0 to 1.4 cases per 100,000 population, with JEV responsible for 52% of cases, and with a decreasing trend over the 10-year period of study [21]. The absence of JEV detection in our study may also have resulted from geographical and seasonal factors. Indeed, most cases of Japanese encephalitis are reported from the Northwest provinces of Vietnam, including Lai Chau, Son La and Dien Bien and from two Northeast provinces (Binh Duong and Bac Lieu), while Thai Binh is less affected [21]. Nevertheless, in a survey addressing acute encephalitis syndrome conducted in Thai Binh from 2004 to 2013, JEV infections accounted for 41% of cases. However, the disease peak was reported from May to July [22], while the current study was conducted between July and October.

Our results showed that EV was responsible for most cases in the outbreak that occurred in Thai Binh in 2020, in line with previous studies [19,23-25]. EV are known to be the main causative agents of viral CNS infection worldwide, particularly in young patients [26]. In a recent review, EV was involved in 48-95% of paediatric patients with viral meningitis [26].

The typical clinical course of EV-associated meningitis is a biphasic fever, accompanied by neurological symptoms during the second febrile peak. Non-specific signs in patients with EV-associated meningitis, including nausea, vomiting, headache, cutaneous rash, and respiratory symptoms, were found in all age groups. In older children, neck stiffness and photophobia are frequent, while neonates often present with irritability, lethargy, and bulging fontanelles. Increased polymorphonuclear leukocytes are a typical sign at the early stages of disease in the CSF laboratory examination [27].

Currently, there are more than 100 genotypes of EV identified, subdivided into 4 species (A, B, C, and D), CV-A5, CV-A7, CV-A9, CV-A16, CV-B2-5, E-4, E-6, E-9, E-11, E-14, E-16, E-25, E-30, E-31, and EV-A71 have been demonstrated to cause meningitis [28]. There is a large variation in mortality between different outbreaks, possibly related to differences in the enterovirus genotype. Apart from EV-A71, the short-term prognosis of meningitis due to EV is favourable [23]. In our study, we identified three different Echovirus types, including mostly E-4, some E-30, majoritarily, and E-6 (in 1 case). This indicated that the rise of central nervous system infections in Thai Binh in 2020 were due to different epidemics. In a study by B’Krong et al., the authors reported the EV serotypes in patients with CNS and respiratory infections in Vietnam from 1997 to 2010 [29]. A total of 51 children and 28 adults were included, with 26 different serotypes of the four EV species (A-D) identified, including EV-D68 and EV-A71. Their results also showed that EV B was associated with viral meningitis in children and adults [29].

Regarding treatment, almost all patients were treated with antibiotics, while bacteria were detected in 2 cases in the CSF and in 4 cases in the blood. It is unlikely that antibiotherapy before admission accounted for this result, given that only 12.1% children received it and that no statistical association was evidenced. It cannot be excluded that the relative lack of capacity for microbiological testing at the hospital may have played a role. In any case, systematic antibiotic treatment in CSN infections in children is not recommended, and antibiotic overuse is an important contributor to the development of antibiotic resistance worldwide [30].

Our study has some limitations. This is a monocentric retrospective study, so it may not represent the entire country. Moreover, most cases were related to an outbreak. Also, due to the lack of microbiological testing facilities in Thai Binh Pediatric Hospital, blood culture was performed in only a small proportion of patients. Based on these results, point-of-care tests, including real-time PCR assays to identify common pathogens in CNS infections, should be implemented for more accurate diagnosis and more appropriate antibiotic use.

## Supplementary Material

Supplemental Material
