# Supplementary material for: Outbreak of central nervous system infections among children in Thai Binh, Viet Nam
Source: Emerg Microbes Infect. 2022 Jun 20;11(1):1683–92. doi: 10.1080/22221751.2022.2088405 (PMC9225704; doi:10.1080/22221751.2022.2088405)
Supplement: Supplemental Material [file TEMI_A_2088405_SM8778.docx]

Supplementary table 1: Clinical presentation among patients infected with different pathogens

| Characteristics | Patients infected with Echovirus 4 virus | Others* | P-value** |
| --- | --- | --- | --- |
| Age |  |  |  |
| <12 months | 6 (13.3) | 20 (37.0) | 0.003 |
| 1 - 4 years | 8 (17.8) | 15 (27.8) |  |
| ≥ 5 years | 31 (68.9) | 19 (35.2) |  |
| Gender |  |  |  |
| Male | 23 (51.1) | 35 (64.8) | 0.17 |
| Female | 22 (48.9) | 19 (35.2) |  |
| Symptoms |  |  |  |
| Fever | 45 (100) | 54 (100) | NA |
| Vomiting | 31 (68.9) | 21 (38.9) | 0.003 |
| Diarrhea | 0 (0) | 2 (3.7) | 0.50 |
| Reduced feeding/eating & drinking | 0 (0) | 3 (5.6) | 0.25 |
| Lethargy | 8 (17.8) | 10 (18.5) | 0.92 |
| Headache (N = 50)^31,19^ | 29 (93.6) | 17 (89.5) | 0.61 |
| Seizure | 0 (0) | 2 (3.7) | 0.50 |
| Physical signs |  |  |  |
| Stiff neck | 25 (55.6) | 20 (37.0) | 0.07 |
| Altered/reduced consciousness | 0 (0) | 3 (5.6) | 0.25 |
| Focal neurological symptoms | 1 (2.2) | 1 (1.9) | 1.0 |
| Irritability (N = 50) ^31,19^ | 2 (4.4) | 4 (7.4) | 0.69 |
| Bulging fontanelle (N = 26)^6,20^ | 1 (16.7) | 2 (10.0) | 1.0 |
| Purpuric rash | 1 (2.2) | 0 (0) | 0.46 |
| Laboratory findings |  |  |  |
| Blood examination |  |  |  |
| White blood cells |  |  |  |
| Normal | 1 (2.2) | 4 (7.4) | 0.37 |
| Elevated | 44 (97.8) | 50 (92.6) |  |
| CRP |  |  |  |
| Normal | 10 (22.2) | 20 (37.0) | 0.11 |
| Elevated | 35 (77.8) | 34 (63.0) |  |
| CSF laboratory examination |  |  |  |
| WBC |  |  |  |
| <10 | 1 (2.2) | 15 (27.8) | <0.0001 |
| 10 - <100 | 10 (22.2) | 15 (27.8) |  |
| ≥ 100 | 34 (75.6) | 24 (44.4) |  |
| Protein |  |  |  |
| ≤ 1.0 g/L | 42 (93.3) | 45 (83.3) | 0.13 |
| >1.0 g/L | 3 (6.7) | 9 (16.7) |  |
| Glucose |  |  |  |
| ≥ 2.2 mmol/L | 43 (95.6) | 51 (94.4) | 1.0 |
| < 2.2 mmol/L | 2 (4.4) | 3 (5.6) |  |

*: Patients infected with other pathogens or testing negative

**: Chi2 or Fisher exact test when appropriate
